# GhostiPy: An Efficient Signal Processing and Spectral Analysis Toolbox for Large Data

**DOI:** 10.1523/ENEURO.0202-21.2021

**Published:** 2021-12-02

**Authors:** Joshua P. Chu, Caleb T. Kemere

**Affiliations:** Department of Electrical and Computer Engineering, Rice University, Houston, Texas 77251-1892

**Keywords:** local field potential, oscillations, signal processing, spectral analysis

## Abstract

Recent technological advances have enabled neural recordings consisting of hundreds to thousands of channels. As the pace of these developments continues to grow rapidly, it is imperative to have fast, flexible tools supporting the analysis of neural data gathered by such large-scale modalities. Here we introduce GhostiPy (**g**eneral **h**ub **o**f **s**pectral **t**echniques **i**n **Py**thon), a Python open source software toolbox implementing various signal processing and spectral analyses including optimal digital filters and time–frequency transforms. GhostiPy prioritizes performance and efficiency by using parallelized, blocked algorithms. As a result, it is able to outperform commercial software in both time and space complexity for high-channel count data and can handle out-of-core computation in a user-friendly manner. Overall, our software suite reduces frequently encountered bottlenecks in the experimental pipeline, and we believe this toolset will enhance both the portability and scalability of neural data analysis.

## Significance Statement

Because of technological innovation, the size of neural recordings has increased dramatically, but downstream analysis code is often not optimized to handle such large scales of data efficiently. Here we have developed GhostiPy, an open source Python package prioritizing performance and efficiency for large data in the context of typical spectral analysis and signal processing algorithms. Users can control hardware resource consumption (e.g., system memory) by setting the level of parallelization and enabling out-of-core processing. Thus, algorithms can be run on a variety of hardware, from laptops to dedicated computer servers. Overall, GhostiPy improves experimental throughput by increasing the portability of analyses.

## Introduction

Advancements in neural recording technologies have enabled the collection of large data in both space (high density/channel count) and time (continuous recordings). During subsequent analysis, the scale of the data induces certain challenges that may manifest as the following scenarios: (1) analysis code takes a long time to complete (high time complexity); and (2) code is unable to complete because of insufficient memory on the hardware (high spatial complexity). Moreover, the scientist may have difficulty finding existing tools that address both 1 and 2 and implement the desired analyses.

Although a potential remedy is to simply upgrade the hardware, it is not an acceptable solution for scientists desiring portability, an important component that improves reproducibility and replicability. In more portable systems, hardware resources may be limited (e.g., using a laptop at the airport). We thus took an alternate approach by efficiently implementing analyses that would trivially scale for different hardware configurations. Our solution is GhostiPy (general hub of spectral techniques in Python), a free and open source Python toolbox that attempts to optimize both time and space complexity in the context of spectral analyses. Methods include linear filtering, signal envelope extraction, and spectrogram estimation, according to best practices. GhostiPy is designed for general purpose usage; while well suited for high-density continuous neural data, it works with any arbitrary array-like data object.

In this article, we first describe the software design principles of GhostiPy to increase efficiency. We then elaborate on featured methods along with code samples illustrating the user friendliness of the software. Finally, we benchmark our software against a comparable implementation, and we discuss strategies for working under an out-of-core (when data cannot fit into system memory) processing context.

## Materials and Methods

An overview of implemented methods can be found in Table 1. Excluding out-of-core support, it is possible to use multiple different packages (OverLordGold Dragon, https://github.com/OverLordGoldDragon/ssqueezepy/; Bokil et al., 2010; Oostenveld et al., 2011; Gramfort et al., 2013; Yegenoglu et al., 2015; Lee et al., 2019; Tadel et al., 2019; Virtanen et al., 2020) to achieve the same functionality. However, the mix-and-match approach can reduce user friendliness since application programming interfaces (APIs) differ across packages and dependency management is more difficult. We believe our unified package provides an attractive solution to this challenge. Table 2 documents the methods currently available in GhostiPy.

**Table 1:** Features implemented by GhostiPy compared with existing software

	Python	Overlap save convolution	Multitaper method	Hilbert transform	CWT	Synchrosqueezed transform	Out-of-core
Ghostipy	+	+	+	+	+	+	+
SciPy	+	+	–	+	–	–	–
Chronux	–	–	+	–	–	–	–
Elephant	+	+	–	+	–	–	–
BrainStorm	+	+	+	+	+	–	–
PyWt	+	–	–	–	+	+	–
Field Trip	–	+	+	+	+	–	–
MNE	+	+	+	+	+	–	–
ssqueezepy	+	–	–	–	+	+	–
MATLAB	–	+	+	+	+	+	–

**Table 2:** Available methods in GhostiPy

Method	Description
analytic_signal()	Compute the analytic signal for a real-valued signal
cwt()	Compute the continuous wavelet transform
estimate_taps()	Estimate number of taps needed for an FIR filter
filter_data_fir()	Filter data using an FIR filter
firdesign()	Design an FIR filter
get_tapers()	Compute DPSS tapers
group_delay()	Get group delay of an FIR filter
mtm_spectrogram()	Use the multitaper method to generate a spectrogram
mtm_spectrum()	Use the multitaper method to estimate a spectrum
plot_fourier_ spectrogram()	Plot a spectrogram generated from a Fourier-based method
plot_frequency_ response()	Plot frequency response of a transfer function
plot_wavelet_ spectrogram()	Plot a spectrogram generated from a wavelet-based method
signal_envelope()	Estimate the envelope of a real- valued signal
signal_phase()	Estimate the instantaneous phase of a real-valued signal
wsst()	Compute the wavelet synchrosqueezed transform

### Software design considerations

As previously noted, successful completion of analyses may be hampered by long computation times or lack of system memory. Specifically, algorithmic time and space complexity is a major determinant for the efficiency and performance of a software method. In general, it is difficult to optimize both simultaneously. For example, time complexity may be reduced by increasing hardware parallelization, at the expense of higher space complexity (memory requirements). While we sought to lower both kinds of complexity compared with existing solutions, we gave space complexity a higher priority. Stated concretely, slow computation time is primarily a nuisance, but failure to complete an analysis because of insufficient memory is catastrophic.

**Figure 1.**
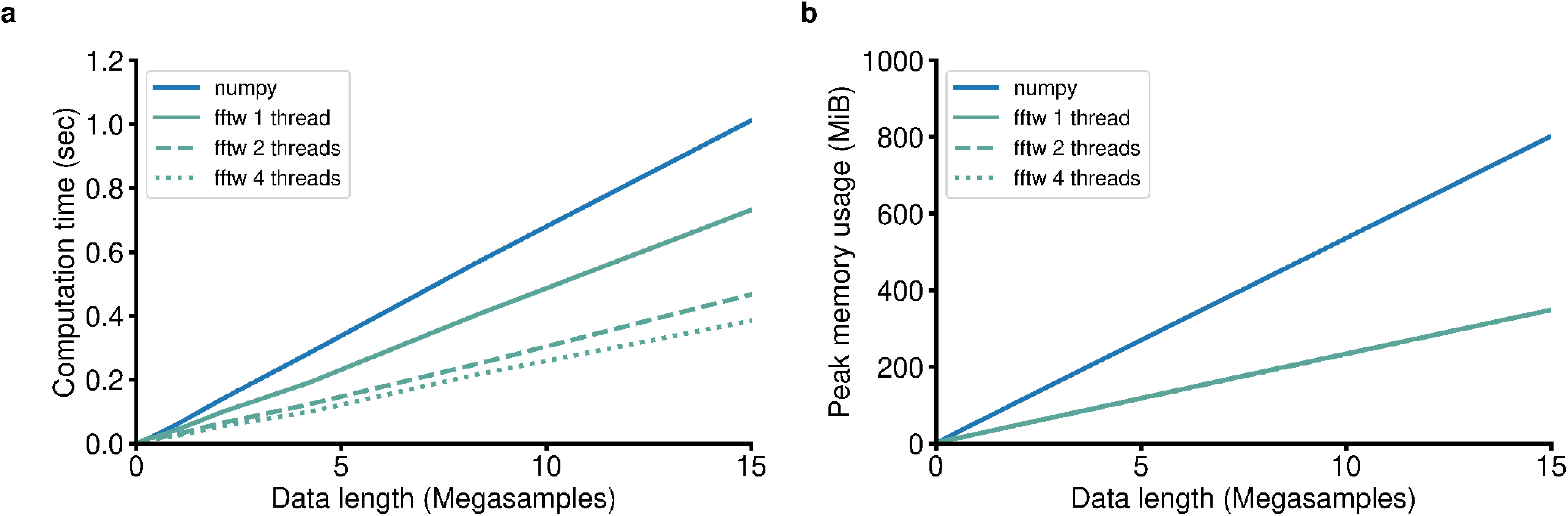
GhostiPy uses fftw rather than numpy for its FFT backend. ***a***, ***b***, Note that when fftw is multithreaded, the computation time can be reduced (***a***) without an increase in memory use (***b***).

Our design decision to prioritize space complexity was particularly critical because it directly influenced which backend library we chose for the fast Fourier transform (FFT), an operation used in the majority of the GhostiPy methods. While investigating the different options, we saw that numpy currently uses the pocketfft backend (https://gitlab.mpcdf.mpg.de/mtr/pocketfft; Van Der Walt et al., 2011). When accelerated with the Intel MKL library, it can be slightly faster than FFTW (https://software.intel.com/content/www/us/en/develop/tools/math-kernel-library/benchmarks.html). However, we have found FFTW (Frigo and Johnson, 1998, 2005) to be superior for memory management and better suited for FFTs of arbitrary length, including prime and odd numbers. An additional benefit of FFTW was its multithreaded capabilities (Fig. 1). We therefore selected FFTW as our FFT backend.

To lower space complexity, we used blocked algorithms, including overlap save convolution, which is not offered in any of the standard Python numerical computing libraries such as numpy or scipy (Van Der Walt et al., 2011; Virtanen et al., 2020). This approach enabled us to process very large data that could not fit in memory (also known as out-of-core processing). Throughout our code, we also used other strategies such as in-place operations.

To lower the time complexity, we used efficient lengths of FFTs wherever possible, and we leveraged modern computing hardware by parallelizing our algorithms. For example, a wavelet transform can be trivially parallelized since the transform for each scale is not dependent on other scales.

### Finite impulse response filter design

GhostiPy provides classical signal processing capabilities such as filtering data, using the efficient overlap save convolution. Filtering data is a ubiquitous operation, but before this stage, the filter must itself be designed. While this step may appear somewhat trivial, it can make a significant difference, including the very existence of theta–gamma phase amplitude coupling (Canolty et al., 2006; Dvorak and Fenton, 2014).

Existing packages such as scipy and MNE offer a variety of finite impulse response (FIR) filter design methods (Gramfort et al., 2013; Virtanen et al., 2020). However, some methods suffer from the following issues. (1) Using the least-squares method, a solution may result in a filter with a magnitude response effectively of zero throughout. This situation is more common when designing filters with passband relatively low compared with the sampling rate. (2) Using the Remez exchange method, the algorithm may simply fail to converge. (3) Using the window method, the transition bands cannot be controlled exactly, and optimality cannot be defined, as is the case for the least-squares (L2 optimal) and Remez exchange (L1 optimal) methods.

Therefore, the GhostiPy filter design uses the method defined in the study by Burrus et al. (1992) for the following reasons: (1) it is simple to design, and the computational complexity is similar to that of a window method and can be implemented on embedded hardware if desired; (2) optimality can be defined, as it is optimal in the L2 sense; (3) transition bands can be defined exactly, and the steepness of the passband rolloff can be controlled by the spline power parameter; and (4) the filter impulse response can be defined analytically. Consequently, its computation does not suffer from the failure modes of the least-squares or Remez exchange methods, as those must solve systems of linear equations. In other words, the design process is reliable and stable.

This method designs a low-pass filter according to the following:

(1) $$h(n)=\frac{\text{sin}(\omega_{0}n)}{\pi n}{\left[\frac{\text{sin}(\Delta n/p)}{\Delta n/p}\right]}^{p}$$

(2) $$\omega_{0}=\frac{\omega_{2}+\omega_{1}}{2},\;\Delta =\omega_{2}-\omega_{1},$$

where *ω*_1_ and *ω*_2_ are radian frequencies defining the transition-band boundaries.

**Figure 2.**
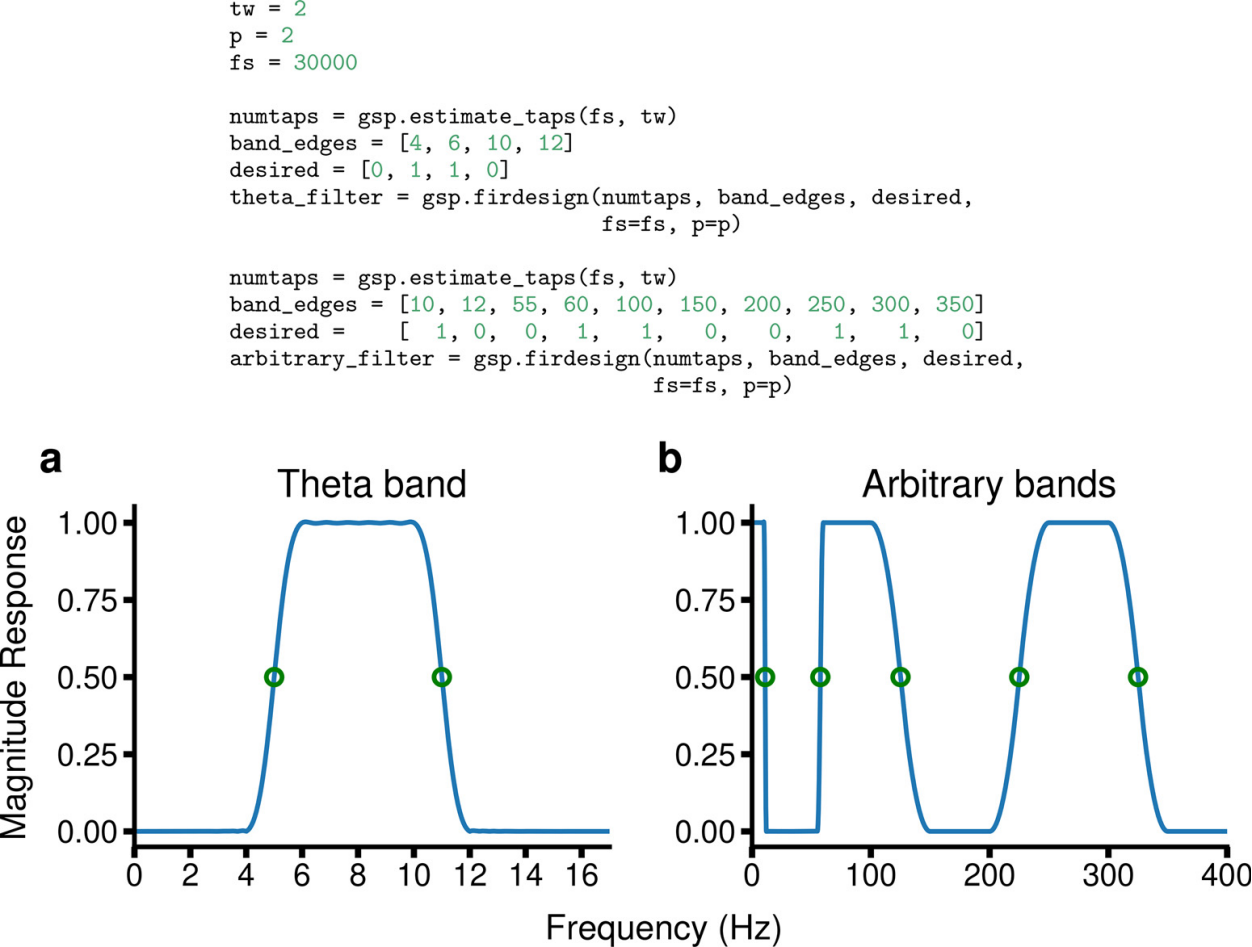
FIR filter design. ***a***, A theta-band filter designed for full bandwidth data. The specification of the transition bands allows for easy determination of critical frequencies. The −6 dB points are exactly the midpoints of the transition bands. ***b***, Filters with arbitrary pass and stop bands may also be designed.

GhostiPy uses the low-pass filter defined in Equation 1 as a prototype to design more complicated filters. As a result, users can request filters with arbitrary magnitude response. An example is shown in Figure 2.

### Multitaper method

Users often wish to perform a spectral decomposition on a signal of interest. This can be accomplished by using the multitaper method (Thomson, 1982; Percival and Walden, 1993). The technique is well suited to reduce the variance of a spectrum estimate, which is particularly useful when working with noisy neural data. The spectrum estimate is obtained as an average of multiple statistically independent spectrum estimators for a discrete signal, *x*[*n*], with sampling frequency *f_s_*, as follows:

(3) $${\hat{S}}_{W}^{mt}(k)=\frac{1}{L}\sum_{l=1}^{L}{\hat{S}}_{l,W}^{mt}(k)$$

(4) $${\hat{S}}_{l,W}^{mt}(k)=\frac{1}{f_{s}}\sum_{n=0}^{N-1}v_{l,W}[n]x[n]e^{-2\pi jkn/N}.$$

Given the length of data *N* and a smoothing half-bandwidth *W*, the tapers *v_l_*_,_*_W_*[*n*] are computed by solving for vectors that satisfy the energy and orthogonality properties, as follows:

(5) $$\sum_{n=0}^{N-1}v_{l,W}[n]v_{l,W}[n]=1$$

(6) $$\sum_{n=0}^{N-1}v_{l,W}[n]v_{m,W}[n]=0,l\neq m.$$

**Figure 3.**
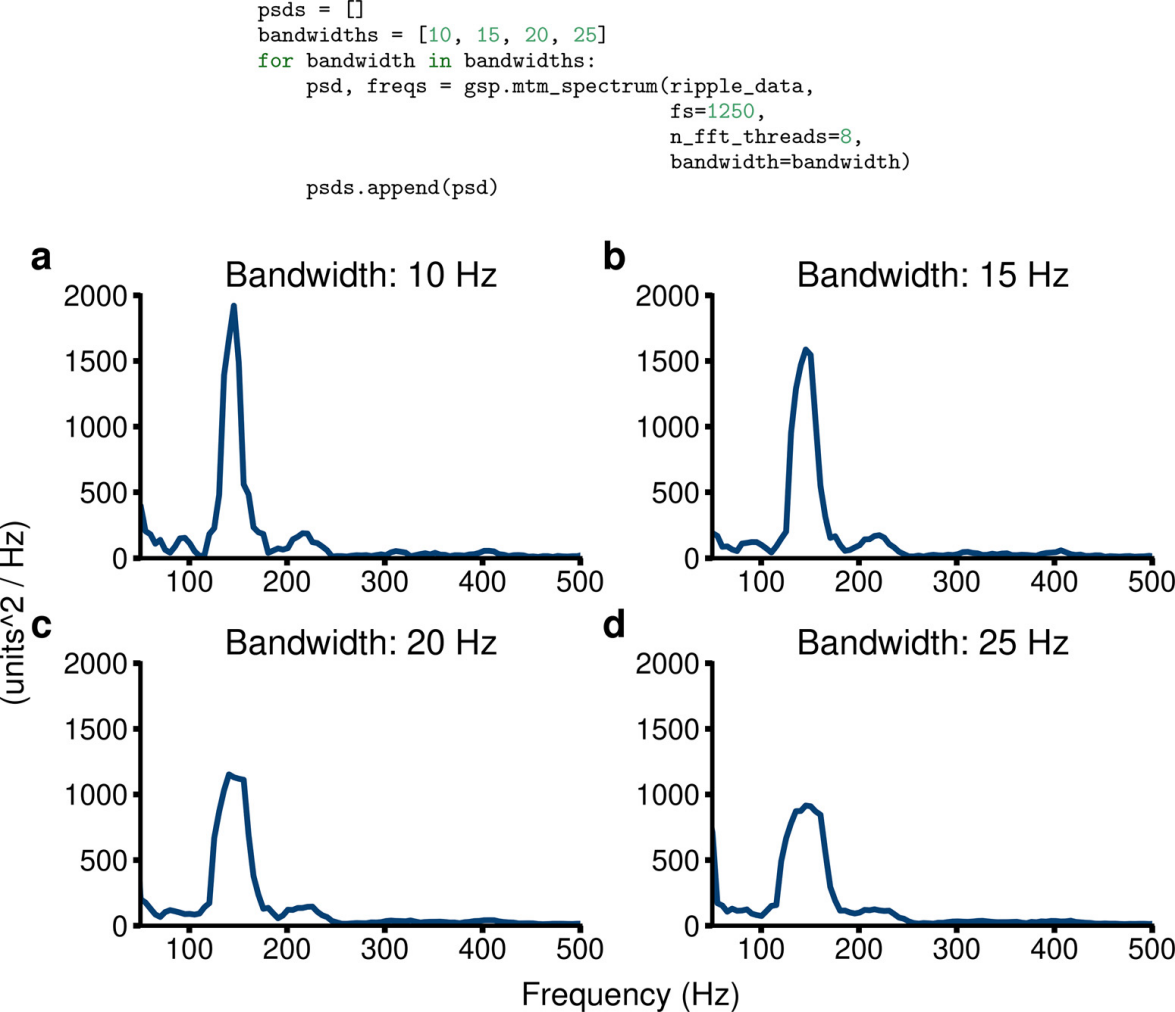
Multitapered spectra. Data are from a sharp wave ripple event, where energy occurs mainly between 100 and 250 Hz. ***a–d***, Bandwidths are 10 Hz (***a***), 15 Hz (***b***), 20 Hz (***c***), and 25 Hz (***d***). Note in the code that the data-sampling rate is 1250 Hz, the FFT is parallelized across eight threads, and ripple_data are a 1D numpy array.

For the tapers, GhostiPy uses the discrete prolate spheroidal sequences (DPSSs), which satisfy Equations 5 and 6 and maximize the power in the band [– *W*,*W*] (Thomson, 1982). An example for computing the multitapered spectrum is shown in Figure 3.

### Continuous wavelet transform

Neuroscientists often use a continuous wavelet transform (CWT) to study transient oscillatory activity. The CWT itself is defined in the time domain by the following:

(7) $$W(a,b)=\int_{-\infty }^{\infty }\frac{1}{\sqrt{a}}\psi^{*}\left(\frac{t-b}{a}\right)x(t)dt,$$

where *ψ*(…) is the mother wavelet function. The transform represents a two-dimensional decomposition in the scale (*a*) and time (*b*) planes. In the frequency domain, the CWT is given by the inverse Fourier transform of the following:

(8) $$W(a)=X(\omega )\Psi^{*}(a\omega ),$$

for a given scale (*a*), where *X* and Ψ are the Fourier transforms of *x* and *ψ*, respectively.

Many mother wavelet functions have been investigated in the literature, but we have focused on the analytic wavelets, as they are found to be superior, particularly for estimating the phase (Olhede and Walden, 2002; Lilly and Gascard, 2006; Lilly and Olhede, 2009, 2012. We have implemented the analytic Morse, Morlet, and Bump wavelets, whose respective frequency domain definitions are as follows:

(9) $$\Psi (a\omega )=2e^{-{\left(a\omega -\omega_{0}\right)}^{2}/2}H(\omega ),$$

(10) $$\Psi (a\omega )=2{\left(\frac{e\gamma }{\beta }\right)}^{\frac{\beta }{\gamma }}{\left(a\omega \right)}^{\beta }e^{-{\left(a\omega \right)}^{\gamma }}H(\omega )$$

(11) $$\Psi (a\omega )=2e^{1-{\left(1-{\left(\frac{\mu -\sigma }{a}\right)}^{2}\right)}^{-1}}1_{(\mu -\sigma )/a,(\mu +\sigma )/a},$$

where $1_{(\mu -\sigma )/a,(\mu +\sigma )/a}$ is the indicator function for the interval (*μ* – *σ*) /*a* ≤ *ω* ≤ (*μ* + *σ*) /*a* and *H*(*ω*) is the Heaviside step function. In our implementation, we use Equation 8 to compute the CWT.

Note that in practice the timeseries *x*(*t*) is sampled, and the CWT is likewise sampled. Then Equation 8 becomes a pointwise complex multiplication of discrete Fourier transforms, where the discretized angular frequencies *ω_k_* are determined by the following:

(12) $$\omega_{k}=\frac{2\pi k}{N\Delta t},$$

where *N* is the number of data samples and Δ*t* is the sampling interval.

A naive implementation of the wavelet transform (Eq. 8) calculates untruncated wavelets the same length as the input data. This is often inefficient because it is equivalent to convolving the data with a time-domain wavelet, mainly consisting of leading and trailing zeros. In our approach, we exploit the fact that wavelets are finite in time and frequency, and we use an overlap-save algorithm to compute the CWT purely in the frequency domain. Note that the latter point is particularly critical: because of the Gibbs phenomenon, using any time-domain representation of the wavelet may violate numerical analyticity for wavelet center frequencies near the Nyquist frequency. It is therefore necessary to use only the frequency domain representation of the wavelet. While we offer both traditional/naive and blockwise convolution implementations, the latter will give superior performance for longer-duration data. We believe that this is a valuable option for researchers and that this is the first tool that uses blockwise convolution to implement the CWT.

For electrophysiological data, a typical wavelet analysis will require computing Equation 8 for 50–500 scales. This is an obvious candidate for parallelization since the wavelet transform for each scale can be computed independently of the others. We use a backend powered by Dask to carry out the parallelization (Rocklin, 2015). Users can set the number of parallel computations to execute and thereby leverage the multicore capabilities offered by modern computing hardware.

### Synchrosqueezing transform

One disadvantage of the wavelet transform is that its frequency resolution decreases as the temporal resolution increases. Strictly speaking, the CWT results in information contained in the (time, scale) plane, but a single frequency is typically assigned to each scale. Regardless, spectral smearing can be observed at higher frequencies/lower scales. However Daubechies (1996) and Thakur et al. (2013) showed the synchrosqueezing transform (SST) could mitigate this issue by transferring a CWT (time, scale) plane information to the (time, frequency) plane.

The synchrosqueezing transform proceeds as follows. For every scale *a*: compute the CWT *W*(*a*) using Equation 8, compute the following partial derivative:

(13) $$\partial_{b}W(a)=j\omega X(\omega )\Psi (a\omega ),$$

and compute the following phase transform:

(14) $$\omega_{f}(a)=\frac{1}{2\pi }ℑ\left(\frac{\partial_{b}W(a)}{W(a)}\right).$$

The phase transform contains the real frequencies each point in the CWT matrix should be assigned to. In practice, the real frequency space is discretized, so the CWT points are assigned to frequency bins. Note that multiple CWT points at a given time coordinate, *b*, may map to the same frequency bin. In this situation, a given frequency bin is a simple additive accumulation of CWT points.

Note the similarity of the SST to the spectral reassignment algorithms in the studies by Gardner and Magnasco (2006) and Fitz and Fulop (2009). However, an important distinction is that the SST only operates along the scale dimension. In addition to preserving the temporal resolution of the CWT, this makes SST data easy to work with since uniform sampling can be maintained.

**Figure 4.**
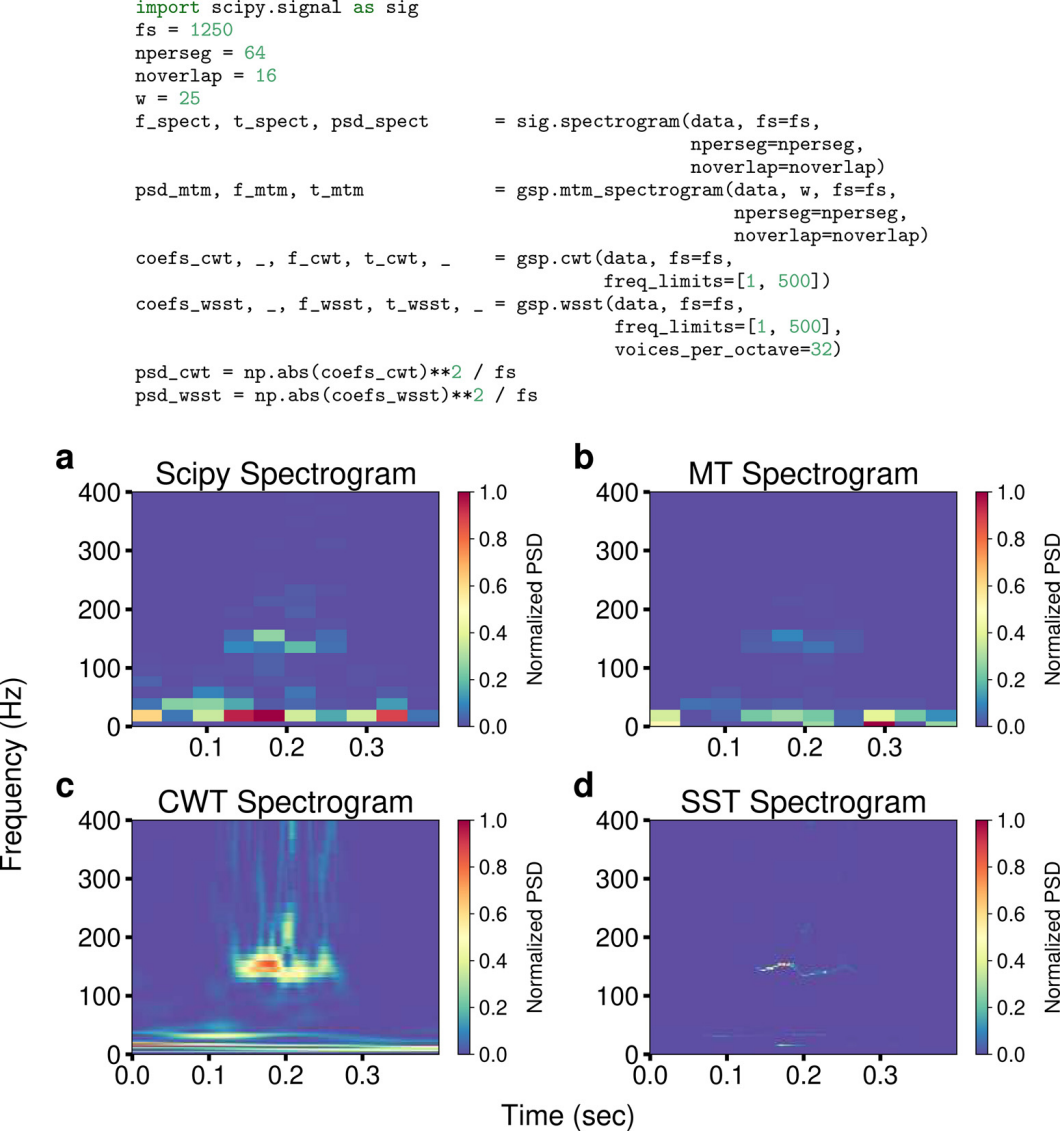
Time–frequency decompositions. ***a–c***, Users can leverage the scipy spectrogram (***a***) along with the methods of GhostiPy (***b–d*)** for a richer understanding of their data. The synchrosqueezed transform in ***d*** gives the overall sharpest time and frequency resolution. Note in the code that data are a 1D numpy array, fs is the sampling rate, nperseg is the spectrogram window size in samples, noverlap is the number of samples overlapping in adjacent windows, and w is the bandwidth for the multitapered spectrogram.

Overall, the spectrogram methods implemented by GhostiPy give an experimenter a more complete picture of the time-varying spectral content of neural data. Figure 4 illustrates this using the scipy standard spectrogram method along with the GhostiPy methods.

### Data availability

The code/software described in the article is freely available online at https://github.com/kemerelab/ghostipy/. Jupyter Notebook, which can be found at https://github.com/kemerelab/ghostipy/tree/master/examples/2021paper, was used to produce the figures. The code and notebooks are also available as Extended Data 1 and Extended Data 2. All results were obtained on an Intel Core i7-4790 desktop computer running the Ubuntu 16.04 operating system.

10.1523/ENEURO.0202-21.2021.ed1 Extended Data 1 Ghostipy-0.2.0. Download Extended Data 1, ZIP file.

10.1523/ENEURO.0202-21.2021.ed2 Extended Data 2 Example data analysis notebooks. Download Extended Data 2, ZIP file.

## Results

### Example analyses

**Figure 5.**
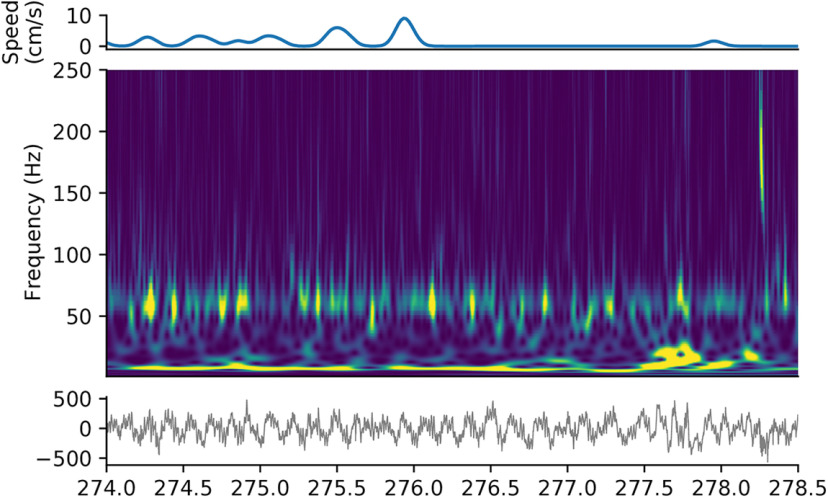
CWT spectrogram of LFP. Spectrogram of local field potential recordings from area CA1 of the hippocampus of a rat during a 5 min exploration (middle), with movement speed (top) and the raw electrophysiological signal (bottom). A number of features of the hippocampal rhythms can be noted in this example, including the pervasive theta oscillation (∼8 Hz), theta-nested gamma oscillations (∼60 Hz) during movement, and, toward the end, a sharp wave ripple (∼200 Hz). Morse wavelets (*γ* = 3, *β* = 10) were used, and frequencies were limited to [1, 250] Hz.

An example spectrogram of local field potentials recorded in area CA1 of the rat hippocampus is depicted in Figure 5. Clearly apparent are the theta oscillation, theta-nested gamma oscillations, and a sharp wave ripple, which occurs after the animal has stopped moving.

**Figure 6.**
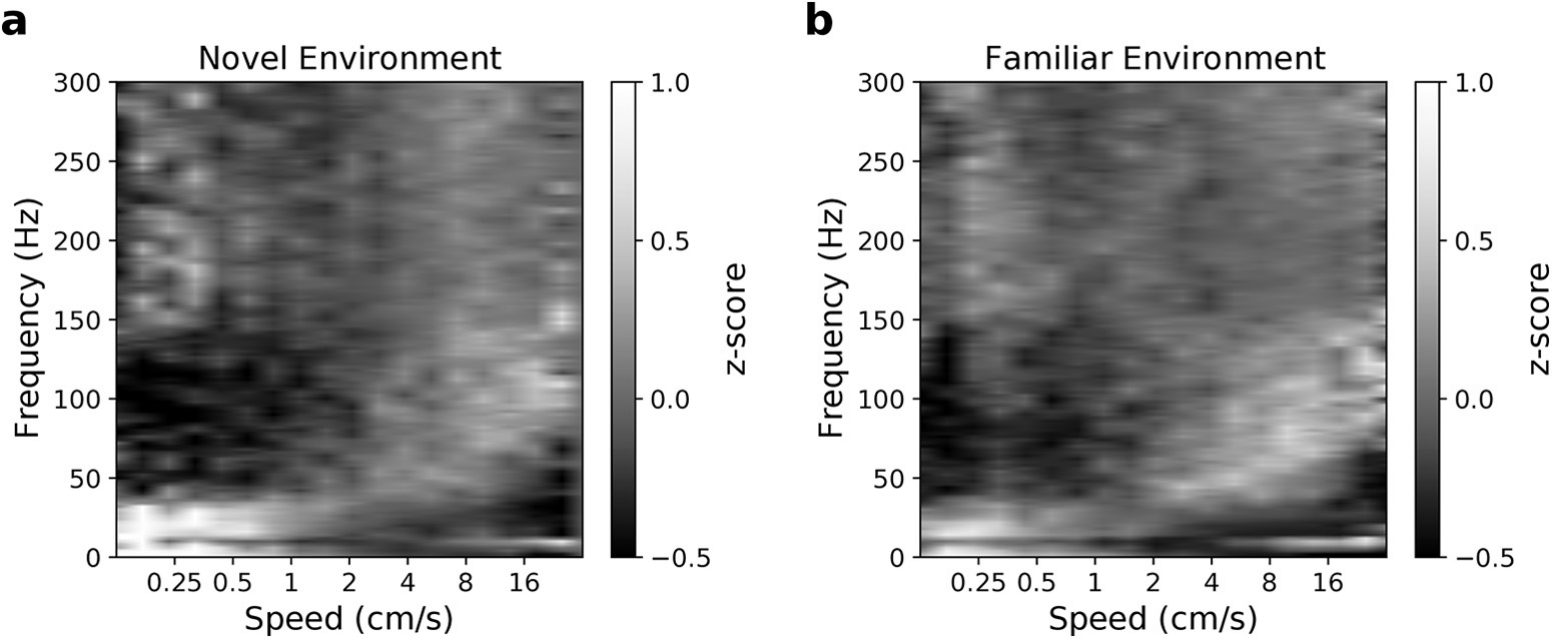
Speed spectrogram. The multitaper spectrogram (bandwidth, 5 Hz) was computed with GhostiPy for nonoverlapping 0.5 s time bins and then *z* scored for each frequency. Each time bin in the spectrogram was assigned to 1 of 21 logarithmically spaced speed bins spanning 0.125–64 cm/s. ***a***, ***b***, The mean PSD for each speed bin is shown for an animal exploring a novel environment (***a***) and a familiar environment (***b***).

**Figure 7.**
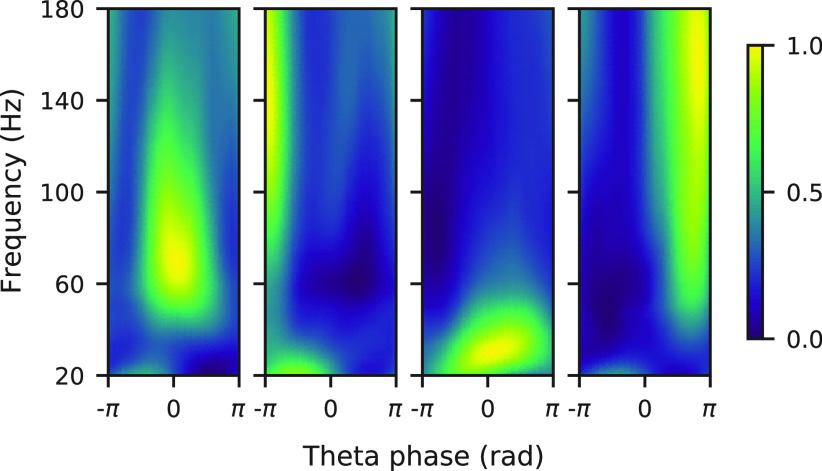
Theta cycle clustering. The Morse wavelet (γ = 3, β = 20) CWT was computed with GhostiPy using 81 frequencies and was subsequently divided into multiple windows, where one window corresponded to one theta cycle. Each CWT sample in a window was assigned to 1 of 20 phase bins according to the instantaneous theta phase at that particular sample. The result was frequency–phase power profiles, which were then clustered into four clusters. Shown is the mean frequency–phase power profile for each cluster. As in Zhang et al., 2019, these use the hc-11 dataset from CRCNS.org (Grossmark and Buzaki, 2016, Grossmark et al., 2016).

In addition, GhostiPy can be used as an intermediate for a multistep analysis. Figure 6 replicates the speed spectrogram analysis in the study by Kemere et al. (2013) for an animal exploring a novel and a familiar environment (Mattias et al., 2015). Figure 7 implements the clustering of theta cycles (Zhang et al., 2019) with Morse wavelets. We have also included notebooks to replicate these example analyses.

### Performance and complexity

**Figure 8.**
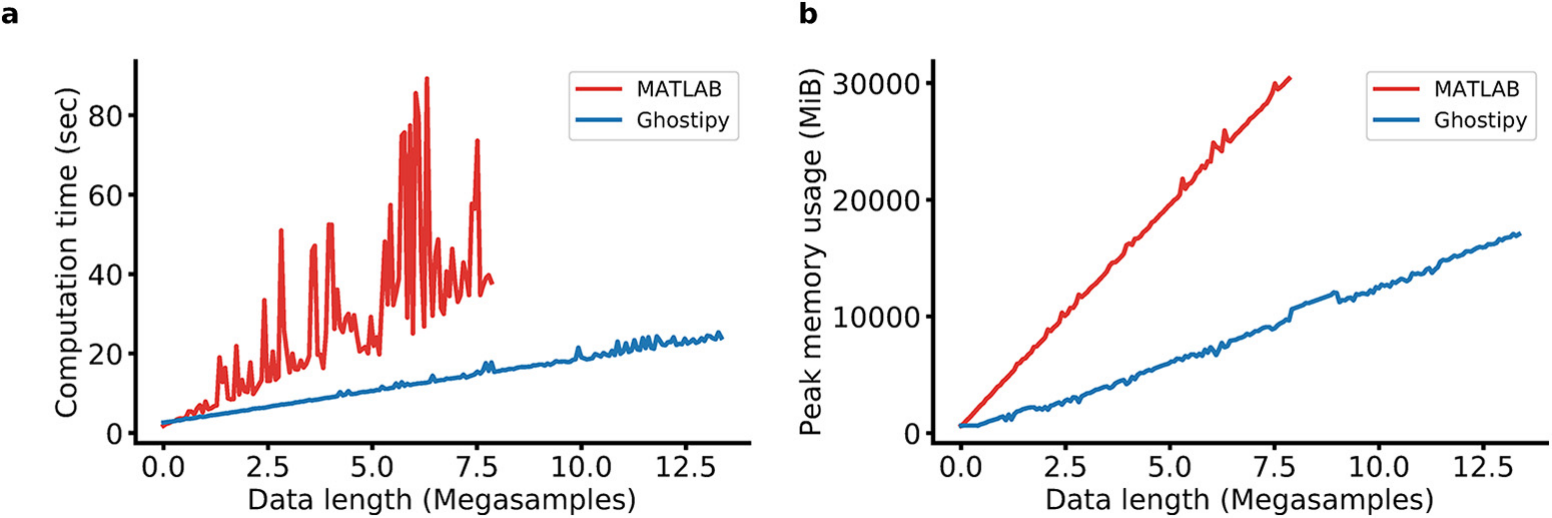
CWT implementation performance. ***a***, ***b***, Our implementation of the Morse continuous wavelet transform outperforms MATLAB in both time (***a***) and space complexity (***b***). Note that MATLAB was unable to complete execution for the full range of the test parameter (data length) because of out-of-memory exceptions. The test machine was an Intel Core i7-4790 (eight hyperthreads) equipped with 32 gigabytes of RAM. In both cases, the CWT was parallelized over available CPU threads.

The calculation of the CWT is computationally intensive and consequently a good method to benchmark performance. Of the software packages listed in Table 1, only MATLAB offered an equivalent solution. It was thus chosen as the reference to compare our implementation against. Figure 8 shows that our implementation results in faster computation times and better memory usage.

It is not entirely clear what accounts for the higher jaggedness in the MATLAB curves from Figure 8. A possible explanation is that the FFT computation is less efficient for an odd-length transform, but the magnitude of the spikes in the curve is surprising given that the FFT backend of MATLAB also uses FFTW. Regardless, we have demonstrated that our implementation is able to achieve lower time and space complexity. When using the functionality offered by GhostiPy, the following three primary scenarios arise with regard to the sizes of data involved in the processing: (1) both the input and output data fit into core memory; (2) the input fits into core memory, but the output does not; and (3) neither the input nor the output fit.

**Figure 9.**
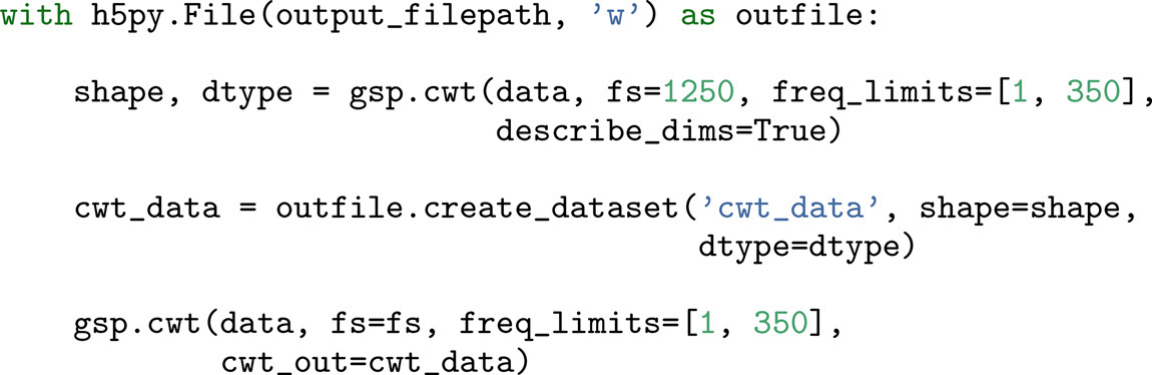
CWT out-of-core. Example code when the output array is too large for main memory. The CWT method is first executed as a dry run to compute the necessary array sizes. Here data are a 1D numpy array, and cwt_data are an HDF5 array created to store the results to disk.

In all of the previous examples, we have restricted ourselves to case 1. However, with the ever-increasing sizes of data, the other cases will inevitably be encountered. Case 2 may arise when attempting to generate spectrograms. As the input is a single channel, memory constraints are rarely an issue. For example, even a 10 h local field potential (LFP) recording sampled at 1 kHz and saved as 64 bit floating point values will require <300 mebibytes (MiB) of memory. However, the size of a wavelet spectrogram computed from these data will be directly proportional to the number of scales/frequencies. For a typical range of 1–350 Hz at 10 voices per octave, this amounts to a space requirement of 85 times that of the input data. Given that this can well exceed the core memory size of a machine, the GhostiPy CWT routine can also accept a preallocated output array that is stored on disk (Fig. 9).

**Figure 10.**
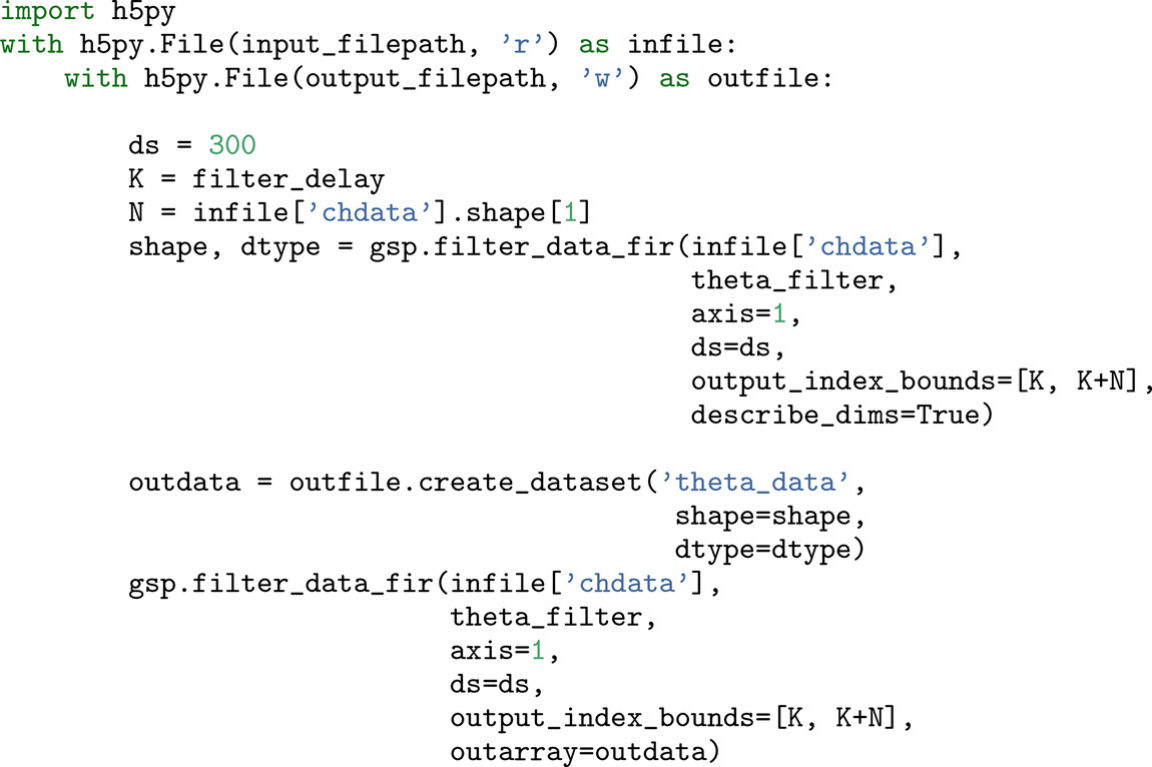
Filtering out-of-core. Filtering data from a large array stored on disk and likewise storing the output on disk. Similar to the CWT out-of-core features, the method is called once as a dry run to compute array sizes, which the user can then pass in to store the result. The filtering method also allows to correct for the delay of the filter and to downsample without storing any intermediate results. Although the example uses the h5py library, any object that behaves like an array can be used. Here ds is the downsampling factor, K is the filter group delay, N is the number of samples, infile[’chdata’] is a (n_channels, n_samples) array, and outdata is an HDF5 array.

Case 3 may arise when a user wishes to filter many channels of full bandwidth data. One case used is a 1 h recording for a 256-channel probe sampled at 30 kHz and stored as a 2 byte signed integer type; already this requires 51 gibibytes. Our strategy is similar to case 2, where an output array is allocated and stored on disk. As for the input, it is read in chunks, and the size of these can be chosen to lower memory usage, although potentially at a cost to computation time. The code in Figure 10 illustrates an example.

Several points can be made about the scheme in Figure 10. Our method allows for downsampling during the convolution, which can reduce the number of stages in a computational scheme. Given full bandwidth data, a traditional strategy to filter to the theta band would look like the following: (1) apply an antialiasing filter; (2) downsample to obtain LFP; (3) store the LFP to disk; (4) apply a theta-band filter; (5) downsample this output; and (5) save the result.

Using the GhostiPy method, it is not necessary to generate the intermediate LFP. To our knowledge, we do not know of other software that allows out-of-core filtering and downsampling in a single function call. The result is a simultaneous reduction in time and space complexity, by storing only the downsampled result and by filtering only once. Filtering to the theta band is now simplified to the following steps: (1) apply a theta filter to the full bandwidth data; (2) downsample the result; and (3) save the result to disk.

## Discussion

We have described the key features of GhostiPy and given examples of its ease of use to perform computations efficiently. Users can thus conduct exploratory spectral analyses quickly across a range of parameters while reducing their concerns for running out of memory, especially since out-of-core computation is supported for many of the methods. Thus, we believe GhostiPy is well suited to handle the ever-increasing size of experimental data.

In the future, we plan to improve GhostiPy with various enhancements. For example, currently the methods are designed to offer the user a lot of low-level control over areas such as multithreading, and to work with raw array types. However, users may desire a higher-level API. For this reason, we believe it would be a worthwhile endeavor to incorporate our work into frameworks such as NWB (Teeters et al., 2015); this would also facilitate more widespread adoption. There are also other analyses we could implement, including the adaptive multitaper method (Percival and Walden, 1993) and other time–frequency reassignment techniques similar to the synchrosqueezing transform (Daubechies et al., 2016).

Our primary contribution is improving the ease and speed at which data analysis can be conducted by developing user-friendly software implementing efficient algorithms well suited for large data sizes. This point is specifically demonstrated by our ability to outperform existing solutions in space and time complexity, and to run computations even in out-of-core memory conditions, which enables machines with 1–10 s of of GBs of memory to process data on the scale of 10–100 s of GBs and higher. In these ways, we have increased the accessibility of neural data analysis by enabling it to be run on hardware such as a laptop computer, a scenario that often was not previously possible.

Finally, the software we developed has a much larger potential impact than the scope described in this article. Although many of the examples given in this article were specific to extracellular rodent hippocampal data, the functionality we implemented is intentionally generic and applicable to many fields. As an example, our code can easily be adapted for use in real-time processing, whether running on embedded hardware or on a laptop computer in a clinical EEG setting. Given the functionality already developed and the full scope of our work, we are optimistic that GhostiPy can help accelerate modern scientific progress.

## Synthesis

Reviewing Editor: Michaël Zugaro, CNRS, Collège de France, Inserm Decisions are customarily a result of the Reviewing Editor and the peer reviewers coming together and discussing their recommendations until a consensus is reached. When revisions are invited, a fact-based synthesis statement explaining their decision and outlining what is needed to prepare a revision will be listed below. The following reviewer(s) agreed to reveal their identity: Adrien Peyrache, Francesco Battaglia.

The manuscript presents GhostiPy, a new free software Python package for the analysis of neural data. This provides a simple, unified interface to easily access numerous algorithms commonly used in spectral analysis. It can run on multiple platforms and hardware, and can compute e.g. spectrograms without prior downsampling. Some algorithms have been optimized compared to standard (numpy) implementations. Overall, this is a welcome addition to the field. Although the Reviewers have not raised any major concerns, they have indicated several ways in which the manuscript could be significantly improved. In particular, the sections should be reordered to reflect the order in which analyses are actually performed (e.g. filtering, then spectograms), and more practical details should be provided (e.g. description of input parameters).

Both Reviewers have pointed out that the mathematical presentation of the algorithms (equations, explanation) was unnecessary given that these are standard methods. More relevant to the specific toolbox presented here would be to offer more details about gains in resource usage (computation time, memory load) and richer examples featuring more use cases. In particular, it would be interesting to know more about scalability: for example, one of the reasons FFTW was favored is that it can be multi-threaded, but no example was provided to illustrate how this library performs on a multi-core machine, or whether it is possible (or there are plans) to scale up use of Ghostipy on distributed (cluster) systems.

Another useful recommendation is to include a list of the API methods/functions of the toolbox, so that it may be seen at a glance what is already possible with the package.

Minor notes follow.

Equations 2 and 3 define an orthonormal basis, however in multi-taper analysis (including in Ghostipy) DPSS (or Slepian) sequences are used, which maximizes power in a restricted frequency band. That condition should be added to the description.

L10: Please clarify that portability is important for reproducibility and replicability.

Table 1: It misleading to list “Morse wavelet” as the default wavelet function. For example, Fieldtrip supports Morlet waveforms.

Eq. 1-4: This study describes a software package, not a mathematical method. There is therefore no need for these equations (at least in the main text). This is true also for the other equations in the main text.

In contrast, the following pseudocode showing how to obtain spectrum contains parameters that are not explained (e.g. “n_fft_threads”), and inputs should be clarified (e.g. “asa”)

Eq. 5-10 are redundant (one can assume readers know the details of CWT) unless their presentation relates to the implementation of CWT in the software. These should be removed, or it should be explained if the implementation of CWT was improved in this toolbox.

The goal of Figure 2 is also unclear. Here, spectrograms from different toolboxes are “compared” (i.e. just displayed) which is not the focus of the paper. The comparison should be between the implementation of SST in this software and others, for example MATLAB. How does this software provide an advantage to users to start using it for computing SST?

Figure 2 gain: x-axis should be time, y-axis Frequency and color bar “normalized PSD", unless the study presents spectrograms in a non-traditional manner. Panels a-d are not described in the legend.

L132-133: Quoting other articles is unusual in a methodological paper, and judgements such as “Theta-gamma coupling was thus a serendipitous discovery.” (L139) are not expected in this kind of study.

Figure 4: The study should report the CWT parameters, the length of the example data, the execution time, comparison to other software packages.

L194: It is unclear why the study focuses only CWT for benchmark. Furthermore, it is not clear if parallelization or the GPU computing capabilities of the CWT in MATLAB was used.

## Author Response

Dear Reviewers,

Thank you for your time! We have made numerous changes in response to your suggestions that we think improve the manuscript and will assist it in helping the readers. Please find below, a point-by-point description of the changes that we’ve made.

In particular, the sections should be reordered to reflect the order in which analyses are actually performed (e.g. filtering, then spectograms), and more practical details should be provided (e.g. description of input parameters).

We’ve reordered the sections to be filtering, multitaper spectrum estimation, and finally spectrograms. We have attempted to provide more information about the input parameters in the text as needed.

Both Reviewers have pointed out that the mathematical presentation of the algorithms (equations, explanation) was unnecessary given that these are standard methods. More relevant to the specific toolbox presented here would be to offer more details about gains in resource usage (computation time, memory load) and richer examples featuring more use cases.

We have added a new figure (Figure 1 in the revised manuscript) detailing performance of FFTW vs numpy’s fft. Figures 6 and 7 demonstrate the use of ghostipy in multi-level analyses previously seen in the literature. Note that the code for these examples are given in the repository - we are happy to include this code in the paper, but we would need guidance on the appropriate way to do this.

In particular, it would be interesting to know more about scalability: for example, one of the reasons FFTW was favored is that it can be multi-threaded, but no example was provided to illustrate how this library performs on a multi-core machine, or whether it is possible (or there are plans) to scale up use of Ghostipy on distributed (cluster) systems.

For scalability, the primary focus of ghostipy was to facilitate portability (i.e. so that analyses could still be run on laptops and other smaller scale hardware). However we recognize that researchers may have access to HPC resources, so we have opened up a GitHub issue to implement ghostipy’s algorithms on distributed systems in the future (https://github.com/kemerelab/ghostipy/issues/2). If we find that this is becomes a widely-needed feature, we will work on adding it.

Another useful recommendation is to include a list of the API methods/functions of the toolbox, so that it may be seen at a glance what is already possible with the package.

We have added Table 2 which lists available methods and the corresponding descriptions.

Minor notes:

Equations 2 and 3 define an orthonormal basis, however in multi-taper analysis (including in Ghostipy) DPSS (or Slepian) sequences are used, which maximizes power in a restricted frequency band. That condition should be added to the description.

We have added to the manuscript: “For the tapers, ghostipy uses the discrete prolate spheroidal sequences (DPSS), which satisfy Eqns. 3 and 4 and maximize the power in the band [-W, W].”

L10: Please clarify that portability is important for reproducibility and replicability.

This sentence has been revised to say “Although a potential remedy is to simply upgrade the hardware, it is not an acceptable solution for scientists desiring portability, an important component that improves reproducibility and replicability.”

Table 1: It misleading to list “Morse wavelet” as the default wavelet function. For example, Fieldtrip supports Morlet waveforms.

Table 1 has been revised as “CWT” rather than “Morse CWT.” Entries are likewise updated.

Eq. 1-4: This study describes a software package, not a mathematical method. There is therefore no need for these equations (at least in the main text). This is true also for the other equations in the main text.

We believe the equations provide value to users who want to understand what ghostipy implements (e.g. the kind of filters it designs, how it computes the multitapered spectrum (since different packages do this differently)).

In contrast, the following pseudocode showing how to obtain spectrum contains parameters that are not explained (e.g. “n_fft_threads”), and inputs should be clarified (e.g. “asa”)

An additional sentence to the Fig. 4 caption was added: “Note in the code that the data sampling rate is 1250 Hz, the FFT is parallelized across 8 threads, and ripple_data is a 1d numpy array.”

Eq. 5-10 are redundant (one can assume readers know the details of CWT) unless their presentation relates to the implementation of CWT in the software. These should be removed, or it should be explained if the implementation of CWT was improved in this toolbox.

Text has been added to this section to clarify that we improved the implementation of the CWT by writing a blockwise convolution algorithm in addition to the naive implementation. Using the blockwise algorithm can improve performance and efficiency.

The goal of Figure 2 is also unclear. Here, spectrograms from different toolboxes are “compared” (i.e. just displayed) which is not the focus of the paper. The comparison should be between the implementation of SST in this software and others, for example MATLAB. How does this software provide an advantage to users to start using it for computing SST?

Our objective for Figure 2 (now Figure 4 in the manuscript) was to demonstrate that ghostipy computes various time-frequency decompositions that are not provided by standard Python packages (see Table 1). By combining existing functionality with ghostipy’s, users can have a more complete understanding of their data.

Figure 2 gain: x-axis should be time, y-axis Frequency and color bar “normalized PSD", unless the study presents spectrograms in a non-traditional manner. Panels a-d are not described in the legend.

Figure 2 has been revised accordingly (now Figure 4 in the manuscript)

L132-133: Quoting other articles is unusual in a methodological paper, and judgements such as “Theta-gamma coupling was thus a serendipitous discovery.” (L139) are not expected in this kind of study.

These lines have been removed. The sentence motivating filter design now reads “While this step may appear somewhat trivial, it can make a significant difference, including the very existence of theta-gamma phase amplitude coupling (PAC)"

Figure 4: The study should report the CWT parameters, the length of the example data, the execution time, comparison to other software packages.

The caption has been revised to describe the length of example data, Morse wavelet parameters, and restriction of analyzed frequencies. Since the figure was meant to provide an example of a use case, comparison to other software packages is not described.

L194: It is unclear why the study focuses only CWT for benchmark. Furthermore, it is not clear if parallelization or the GPU computing capabilities of the CWT in MATLAB was used.

We have specifically focused on the CWT to demonstrate the simultaneous reduction in space and time complexity compared to MATLAB. The caption for Figure 5 now includes the statement “In both cases, the CWT was parallelized over available CPU threads.”

We appreciate your reconsideration of the manuscript.

Sincerely,

The authors
